# Pediatric brain tumors in low- and middle-income countries: available evidence on recent advancements in management, challenges, and recommendations – editorial

**DOI:** 10.1097/JS9.0000000000000226

**Published:** 2023-02-16

**Authors:** Andrew A. Wireko, Heli Patel, Aashna Mehta, Riaz Jiffry, Favour T. Adebusoye, Goshen D. Miteu

**Affiliations:** a Sumy State University, Sumy, Ukraine; b Dr. Kiran C Patel College of Allopathic Medicine, Nova Southeastern University, Davie, Florida, USA; c Faculty of Medicine, University of Debrecen, Debrecen, Hungary; d Royal College of Surgeons in Ireland, University of Medicine and Health Sciences, Dublin, Ireland; e School of Bioscience, University of Nottingham, England, UK

*Dear Editor*,

Brain and spinal cord tumors are the second most common type of tumor in children, after leukemia^1^. They typically account for 25% of all childhood cancers, with over 4000 brain and spinal cord tumors diagnosed in children worldwide annually^1^. Pediatric brain tumors (PBTs) are the most common and lethal solid malignancy in children, affecting children of all ages, with males being affected more. PBTs are estimated to occur between 0.3 and 2.9 times per 100 000 children each year^2^. PBTs’ survival rates vary greatly depending on the type and stage of the tumor, as well as access to diagnosis and treatment.

Many advances in the diagnosis and treatment of PBTs have occurred in recent years, improving survival rates, particularly in the majority of the high-income countries (HICs)^3^. While HICs have a wealth of data highlighting the prevalence and advancements in the management of PBT, low- and middle-income countries (LMICs) have a significant scarcity of data on the same issue. PBT has been underreported in LMICs due to poor health care systems and a lack of quality research. When compared to HICs, current data on PBT survival rates in LMICs are extremely low. Surgery is usually the primary treatment for PBTs, but the number of neurosurgeons in many LICs is very scarce and unequally distributed^3^. According to the WHO, only 15–45% of pediatric cancer patients in LMICs receive optimal care that leads to a cure, compared to 80% in developed countries (Fig. 1)^4^.

**Figure 1:**
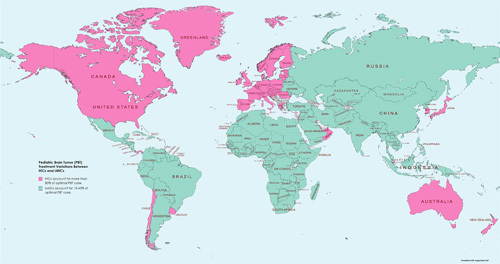
Pediatric cancer treatment variations between high-income countries (HICs) and low- and middle-income countries (LMICs) (created with Mapchart.net)^4^.

Oncological and surgical management have improved in many LMICs in recent years as a result of global efforts. For example, the impact of the ‘My Child Matters’ initiatives was assessed in over 10 different LMICs between 2006 and 2016^5^. Many of their initiatives saw an increase in the percentage of correct diagnoses and effective management as a result of improved health care infrastructure, earlier referral, awareness campaigns, and education for primary care health care staff. Their advanced pathology and other diagnostic services have been critical in increasing the proportion and accuracy of cancer diagnoses, particularly in Morocco, the Philippines, Senegal, and Ukraine^5^. As a result of the establishment of LMIC and HIC twinning programs, there has also been an improvement in technological advancement, diagnosis, and treatment in pediatric oncology. Regional collaborative projects have recently been established in the Middle East, Central and South America, Asia, Africa, and Oceania, with the potential to improve pediatric patients’ access to treatment^6^.

Despite an overall increase in efforts to improve care for children with brain tumors, underdiagnosis, poverty, subpar clinical evaluations, difficulty accessing facilities for multimodal treatment, and a lack of overall higher level care for PBTs have been observed in LMICs^3^. The presence of global variation in the reported case burden of brain tumors, with a higher incidence in HICs than LMICs, is attributed to underreporting in LMICs. This underreporting is due to limited diagnostic capacity, poor research capacity, and poor access to care^7^. Also, the scarcity of advanced surgical techniques such as neuroendoscopy and minimally invasive surgery, for example, is not widely available in LMICs, compounding the problem. Other oncological treatment regimens are also insufficient in LMICs. For instance, a 10-year retrospective study in Pakistan, exemplifies the lack of targeted treatments such as high-dose chemotherapy, as well as major financial hardships that impact pediatric patient care in LMICs^8^. The majority of the 175 patients received one or more types of intervention for intracranial tumors, such as surgery, radiation, chemotherapy, or a combination of all of them^8^. However, 30% of patients who needed a metastatic workup did not receive the necessary treatment for unknown reasons. Furthermore, approximately half of the 175 patients did not return for follow-up due to inadequate socioeconomic support, a lack of coordination among management teams, and a lack of accommodations for patients traveling from distant locations^8^.

LMICs lack well-trained pediatric oncology specialists and other health professionals. The most significant barrier is a lack of access to specialist medical care and resources, as most LMICs have a scarcity of skilled neurosurgeons, radiologists, and other medical personnel capable of caring for PBT patients^9^. Most neuro-oncology specialists and nonphysicians are in short supply, resulting in poor management and an increase in PBT mortality in LMICs when compared to HICs. Aside from understaffing, there is an uneven distribution of qualified oncological, pediatric, surgical, and other health care workers across health facilities, particularly at the primary care level.

Brain tumors are the leading cause of cancer-related death in children in many parts of the world, but data on the incidence, prevalence, and outcomes of these tumors in LMICs is limited. For instance, Herdell *et al* 3 discovered that only 8 of 46 sub-Saharan African (Fig. 2) countries were represented in the scientific literature on PBTs, implying a significant regional difficulty in accessing high-quality pediatric cancer databases and research results. This data scarcity is concerning because it may lead to an inaccurate representation of the region’s PBT prevalence and management gaps. The true burden of PBTs in these underserved areas is unknown, making it difficult to develop effective prevention, diagnosis, and treatment strategies.

**Figure 2:**
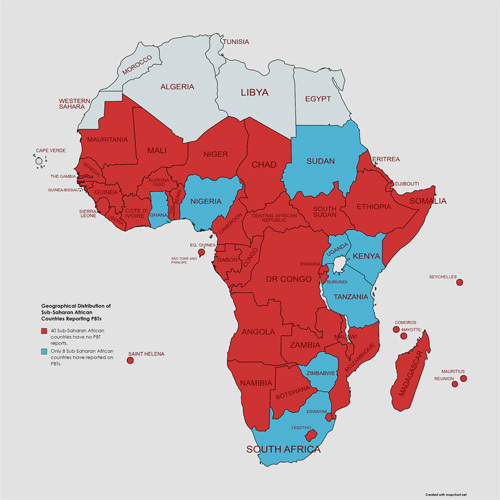
Geographical distribution of Sub-Saharan African (SSA) countries reporting pediatric brain tumor (PBTs), where only 8 out of 48 countries reported on PBTs. Ghana, Nigeria, Sudan, Uganda, Tanzania, Kenya, Zimbabwe, and South Africa were the countries involved (created with Mapchart.net)^3^.

Due to the limitations of effective PBT diagnosis and treatment in LMICs, several steps could be taken to reduce mortality and improve treatment outcomes. It is important that LMICs prioritize the development of infrastructure and resources to provide specialized medical and surgical care for effective PBT management. Building specialized pediatric cancer centers, training health care professionals, and providing access to advanced diagnostic and treatment technologies all need to be prioritized. LMICs should also place more emphasis on diagnostic capacity enhancements to improve the diagnostic and treatment outcomes of PBTs. National health care strategic plans should take oncological care safety, timeliness, and accessibility seriously. Local governments should also focus on raising community awareness of common PBTs, increasing early detection through self-awareness of symptoms, and dispelling the myth that neuro-oncological care is completely unaffordable and futile.

The paucity of literature in the field of PBTs reflects the need for more research opportunities; cross-regional collaborations and partnerships may aid in the establishment of a framework or foundation to improve data collection and analysis in order to better study trends in LMICs and thus provide more targeted care. More research on PBT management in resource-limited settings is needed to help health institutions and policymakers design plans that are tailored to the region’s needs while keeping its resources and limitations in mind.

To address any knowledge gaps that may be contributing to outcome disparities between pediatric populations in LMICs and HICs, it will be critical to investigate the current state and quality of pediatric oncology and surgical medical education and training. While these suggestions are not intended to be exhaustive, they do serve as a jumping-off point for further investigation of other potential solutions.

## Ethical approval and consent to participate

Not applicable.

## Sources of funding

None.

## Authors’ contributions

A.A.W.: conceptualization ideas. All authors were involved in this processes of data curation, writing of initial draft, review and editing, and final approval of the draft.

## Conflicts of interest disclosure

Authors declare no conflict of interest.

## Research registration unique identifying number (UIN)

1. Name of the registry: NA.
2. Unique identifying number or registration ID: NA.
3. Hyperlink to your specific registration (must be publicly accessible and will be checked): NA.

## Guarantor

Andrew Awuah Wireko.

## Availability of supporting data

No new data generated.
